# Efficient Targeted Next Generation Sequencing-Based Workflow for Differential Diagnosis of Alport-Related Disorders

**DOI:** 10.1371/journal.pone.0149241

**Published:** 2016-03-02

**Authors:** Gábor Kovács, Tibor Kalmár, Emőke Endreffy, Zoltán Ondrik, Béla Iványi, Csaba Rikker, Ibolya Haszon, Sándor Túri, Mária Sinkó, Csaba Bereczki, Zoltán Maróti

**Affiliations:** 1 University of Szeged, Faculty of Medicine, Department of Pediatrics and Pediatric Health Center, Szeged, Hungary; 2 University of Szeged, Faculty of Medicine, First Department of Internal Medicine, Szeged, Hungary; 3 University of Szeged, Faculty of Medicine, Department of Pathology, Szeged, Hungary; 4 Péterfy Sándor Hospital Department of Internal Medicine 1, Budapest, Hungary; Odense University Hospital, DENMARK

## Abstract

Alport syndrome (AS) is an inherited type IV collagen nephropathies characterized by microscopic hematuria during early childhood, the development of proteinuria and progression to end-stage renal disease. Since choosing the right therapy, even before the onset of proteinuria, can delay the onset of end-stage renal failure and improve life expectancy, the earliest possible differential diagnosis is desired. Practically, this means the identification of mutation(s) in *COL4A3-A4-A5* genes. We used an efficient, next generation sequencing based workflow for simultaneous analysis of all three *COL4A* genes in three individuals and fourteen families involved by AS or showing different level of Alport-related symptoms. We successfully identified mutations in all investigated cases, including 14 unpublished mutations in our Hungarian cohort. We present an easy to use unified clinical/diagnostic terminology and workflow not only for X-linked but for autosomal AS, but also for Alport-related diseases. In families where a diagnosis has been established by molecular genetic analysis, the renal biopsy may be rendered unnecessary.

## Introduction

Familiar Benign Haematuria (FBH) and Alport syndrome (AS) are familial hematuric diseases which in case of AS regularly escalate to chronic kidney disease (CKD) stage 5 (formerly referred as end stage renal disease). AS patients usually have sensorineural high-tone deafness and ocular abnormalities affecting the lens and fundus [1,2].

Today, more focus has been placed on treating patients early to prevent or delay future end stage kidney damage. Although, the pathogenesis of CKD is multifactorial, some of the suggested therapeutic interventions (anti-hypertensive therapy, glycemic control, anti-proteinuric therapy, renoprotection, and life style management such as restricted protein intake, cessation of cigarette smoking and chronic analgesic-abuse) are encouraging. These preventive steps the more earlier are implemented the more efficient they are [3–5].

There has been an old and ongoing dispute to differentiate between AS and FBH based on the wide spectra of observed clinical symptoms, microscopic analysis of renal biopsy, immunological examination and family history [6]. The first observed clinical signs are postponed by the fact that in the intrauterine life our glomerular basal membrane does not contain *COL4A3-A4-A5*, but *COL4A1* and *COL4A2*. First symptom of AS is hematuria which appears usually 10–15% of cases in the first year of life, while the majority of patients show symptoms only around 5–7 years of age [7]. Because of this late manifestation and similarity between early symptoms of AS and FBH neither clinical nor histological methods are appropriate for early diagnosis. However, the correct genotyping can give an informed clue for the expected progression of the disease, aid the determination of mode of inheritance useful to the family members showing yet no symptoms, and most crucially is unavoidable for kidney donor selection [7,8].

In order to determine the mode of inheritance of AS and/or FBH a pathogenic mutation(s) in the *COL4A5* gene present on X chromosome or mutations in either the *COL4A3* or *COL4A4* genes on chromosome 2 should be found [9]. The most common form of AS, with approximately 4 in every 5 cases, is inherited in an X-linked fashion. X linked carrier females usually show variable intermediate phenotype. Due to imbalances in random X inactivation the phenotype can vary even between family members. In case of FBH, the mode of inheritance is autosomal dominant, and this disease is caused by a single heterozygous mutation either in *COL4A3* or in *COL4A4* genes [10]. If there are two mutations either in *COL4A3* or *COL4A4* genes,—a more severe—form of AS develops. Because of this FBH can be viewed as the carrier state of AS. There are very few reports in the literature, the vast majority of which from the pre-next generation sequencing (NGS) era where autosomal dominant form of AS reported, and only one mutation was found in either the *COL4A3* or *COL4A4* gene. Up to now it is not clear whether this form of AS may only be the result of prior technical limitations or it is real, which can be solved only with sequence-based analysis of larger data set and with the introduction of a new technology [11].

Here we focus on the improvement of genetic diagnosis of type IV collagenopathies, AS and FBH [12]. Sequential (one by one) genetic testing for mutations in *COL4A3-A4-A5* genes has become an integral part of the clinical evaluation. In our previous study on type IV collagenopathies we detected large number (more than a dozen) of non-synonymous variants in each individual in these 3 genes, therefore the distinction between causative mutations and benign variants is crucial [13]. Since all three genes are large (contains 52, 48 and 51 exons, respectively) the use of conventional Sanger sequencing is time-consuming, expensive and can suffer from some technical limitation (such as failing to detect insertion/deletion with certain sizes in a heterozygote subject). One way to overcome these problems is to sequence all three genes simultaneously using NGS [14].

In this paper our aim is three-fold. First, we present a new, efficient, amplicon based NGS protocol for simultaneous analysis of the coding regions (all the exons and flanking intronic sequences) of the *COL4A3*, *A4* and *A5* genes since a previously published NGS-based approach failed to detect mutations in 45% of their cases [11]. Both mutations and polymorphisms in the 3 investigated *COL4A* genes are thought to be highly population-specific due to the lack of selection pressure in case of the polymorphisms and low selection pressure in case of FBH. Thus, in order to further aid the classification of *COL4A3-A4-A5* genetic variations, we present *COL4A3-A4-A5* polymorphisms of 66 unrelated Hungarian non AS/FBH patients’ data obtained by NGS. Finally, we set the task of identifying the genetic causes of 17 Hungarian AS/FBH independent cases, analyzing 17 AS/FBH patients using our NGS panel and Sanger validation of 55 additional family members.

## Materials and Methods

In accordance with Hungarian regulations written informed consent was obtained from each patient in the framework of a genetic counseling session. The study protocol adhered to the tenets of the Declaration of Helsinki and was approved by the regional ethical committee (Ethical board of University of Szeged)

### Patients

Our Hungarian cohort consisted of 3 individuals (I1-I3, where family members were not available for either clinical or genetic analyses) and 14 COL IV nephropathy families (F1-F14) where clinical data and inheritance of clinical symptoms supported Alport/FBH diagnosis while other possible clinical causes were excluded. All volunteer family members underwent urinalysis and renal function evaluation. Kidney biopsy was not performed on all proband. All available clinical and pathological information is summarized in Table 1. After obtaining written informed consent peripheral blood samples were collected from both the affected and unaffected members of the families. We used anonymised DNA samples from our licensed biobank for the 66 Hungarian non AS/FBH control samples. Criteria for FBH: mild persistent haematuria (25–30 RBC/HPF), mild (0.5–1.0 g/day) or no protenuria, first appereance after 10 years of age. Criteria for Alport: persistent haematuria (>30, 50–100 RBC/HPF), persistent protenuria (0.5–5.0 g/day).

**Table 1:** Clinical and pathological details of the investigated Hungarian AS/FBH families.

**Family**	**Individuals**	**Age**	**Hematuria**	**Proteinuria**	**Renal function**	**Biopsy, histology**	**hearing**
**F1**	I/2	36 yrs	microhematuria	none	normal	none	normal
	II/2	3 yrs	persistent hematuria	none	normal	At age 3 could not discriminate between AS or TBMN	normal
**F2**	I/1	58 yrs	none	none	normal	not performed	normal
	II/2	31 yrs	microhematuria	yes	normal	At age of 31 TBMN was suggested	normal
**F3**	I/2	53 yrs	hematuria	none	normal	At age 7: suggested TBMN	ND
	II/1	32 yrs	hematuria	yes	on dialysis	At age 17: suggested TBMN	ND
	II/2	30 yrs	hematuria	0.5 g/day	eGFR 90 (CKD Stage 1), had transplantation and now on dialysis	not performed	ND
**F4**	I/1	38 yrs	microhematuria	no	normal level of se-creatinine	not performed	mild right-side and moderate left-side hipacusis
	II/1	15 yrs	ND	ND	on dialysis since age of 15	not performed	bilateral mild hipacusis
	II/2	13 yrs	hematuria	5 g/day at age of 13		At age of 18 diffusely thin GBM, basket weaving in several loops, α3 chain was entirely absent, α5 chain exhibited very focal expression	ND
	II/3	16 yrs	microhematuria	ND	normal	ND	normal at age of 29
**F5**	I/2	86 yrs	hematuria	ND	ND	ND	ND
	II/3	61 yrs	hematuria	ND	ND	ND	ND
	II/4		hematuria	ND	ND	ND	ND
	III/2	30 yrs	ND	ND	kidney transplantation at age of 31	ND	ND
	IV/1	17 yrs	hematuria	yes	no kidney transplantation at age of 25	ND	normal at age of 25
	IV/2 and IV/3 twins	16 yrs	ND	ND	both had kidney transplantation at age of 16	ND	ND
**F6**	II/1 died at age 13	13 yrs	ND	ND	ND	ND	ND
	II/5	21 yrs	hematuria	yes	ND	At age 11; not conclusive	ND
**F7**	I/1	80 yrs	none	none	se-creatinine normal	not performed	ND
	I/2	49 yrs	10–12 dysmorphic RBC/hpf	yes	se-creatinine normal	At age of 49 diffusely thin GBM, glomerular scars	moderate bilateral hearing loss
	II/2	60 yrs	10–15 dysmorphic RBC/hpf	yes	se-creatinine normal	not performed	ND
	II/4	62 yrs	10–15 dysmorphic RBC/hpf	none	ND	not performed	ND
	III/1	46 yrs	8–10 dysmorphic RBC/hpf	none	se-creatinine normal	At age of 46 diffusely thin GBM	bilateral hearing loss
	III/3	42 yrs	hematuria	yes	ND	not performed	ND
	III/4	40 yrs	hematuria	ND	ND	ND	ND
	III/6	47 yrs	30–35 dysmorphic RBC/hpf	1.5 g/day	se-creatinine normal	At age of 47 diffusely thin GBM, doubled in some loops	bilateral hearing loss
	III/8	44 yrs	hematuria	ND	ND	not performed	ND
	III/9	42 yrs	25–30 dysmorphic RBC/hpf	0.8 g/day	se-creatinine normal	At age of 42 diffusely thin GBM	moderate bilateral hearing loss
	IV/1	17 yrs	8–10 dysmorphic RBC/hpf	3 g/day	se-creatinine normal	At age of 17 diffusely thin GBM, with splitting in some loops	moderate bilateral hearing loss
	IV/2	24 yrs	8–10 dysmorphic RBC/hpf	3 g/day	se-creatinine normal	At age of 24 diffusely thin GBM, with splitting in some loops	bilateral hearing loss
	IV/3	36 yrs	hematuria	ND	ND	not performed	normal
	IV/7	15 yrs	50–60 dysmorphic RBC/hpf	0.8 g/day	se-creatinine normal	At age of 15 diffusely thin GBM	moderate unilateral hearing loss
	V/1	4 yrs	persistent hematuria	ND	ND	not performed	normal at age of 4
**F8**	I/1	38 yrs	hematuria	ND	ND	not performed	normal
	II/1	5 yrs	hematuria	ND	ND	not performed	normal
	II/2	12 yrs	100 RBC/hpf	ND	ND	not performed	normal
**F9**	I/1	71 yrs	hematuria	ND	ND	not performed	ND
	II/2	45 yrs	hematuria	ND	ND	At age of 32 could not confirm either AS or TBMN	ND
	III/1	16 yrs	hematuria	ND	ND	At age of 3 TBMN was suggested	ND
**F10**	II/1	65 yrs	hematuria	ND	ND	not performed	ND
	II/2	67 yrs	hematuria	ND	ND	not performed	ND
	III/2	44 yrs	hematuria	ND	ND	not performed	ND
	IV/1	12 yrs	hematuria	ND	ND	not performed	mild hipacusis
**F11**	I/1	50 yrs	30 RBC/hpf	ND	ND	not performed	ND
	II/1	24 yrs	30 RBC/hpf	yes	ND	At age of 24 secondary FSGS, likely TBMN but could not exclude AS	normal
	II/2	15 yrs	30 RBC/hpf	yes	decreasing eGFR	ND	ND
**F12**	II/1	69 yrs	hematuria	none	ND	not performed	normal
	II/3	71 yrs	hematuria	none	ND	not performed	normal
	II/5	68 yrs	hematuria	none	ND	not performed	normal
	III/3	37 yrs	hematuria	none	ND	At age of 42 TBMN was suggested	normal
	III/6	42 yrs	hematuria	none	ND	not performed	normal
	III/7	35 yrs	hematuria	none	ND	not performed	normal
	III/8	39 yrs	hematuria	none	ND	not performed	normal
**F13**	I/1	38 yrs	hematuria	no	ND	ND	normal
	II/2	6 yrs	hematuria	no	ND	ND	normal
**F14**	I/2	35 yrs	microhematuria	no	ND	not performed	ND
	II/1	2 yrs	microhematuria at age of 3	no	ND	not performed	ND
	**Individuals**	**Age**	**Hematuria**	**Proteinuria**	**Renal function**	**Biopsy, histology**	**hearing**
	I1	35 yrs	hematuria	yes	ND	not performed	ND
	I2	38 yrs	microhematuria	0.8 g/day	ND	not performed	ND
	I3	1 yr	hematuria	none	ND	not performed	ND

RBC—red blood cell; eGFR—estimated glomerular filter rate; CKD—chronic kidney disease; ND—no data; hpf–(microscopic) high power field; FSGS—focal segmental glomerulosclerosis, GBM—Glomerular basement membrane, AS—Alport syndrome, FBH—Familiar Benign Hematuria. There was no data on eye sight problems in the patients.

### Methods

The genomic DNA from peripheral blood leucocytes was isolated by a salting out procedure. In our earlier study we made genetic test to confirm the co-segregation pattern of hematuria with STR markers and LOD score analysis and we performed mutation screening with High Resolution Melting (HRM) analysis and Sanger sequencing of a different set of *COL4A5* families [13]. Here we used an alternative method sequencing coding regions and the flanking splice region of the *COL4A3*, *COL4A4*, and *COL4A5* genes by new generation sequencer. Based on the family tree, initially we choose one proband from each family (proteinuria: >0.5 g/day; hematuria 25–30 red blood cell/high power field (RBC/HPF) for the NGS.

Our AS/FBH panel consisting of all the three *COL4A3-4-5* genes was designed using the Ion AmpliSeq^™^ Designer version 4.2.1 from Life Technologies. Gene enrichment was performed by the AmpliSeq amplicon based target enrichment system. Sample enrichment was performed by the Ion AmpliSeq^(TM)^ Library Kit, Life Technologies. The samples were barcoded and sequenced on IonTorrent 316 chip. We aimed for at least 25 times coverage (more than 90% of the target were covered at least 100 times and more than 95% of the target were covered at least 50 times). Bioinformatical analysis was performed by CLC Bio, Torrent Suite 4.2 and Illumina VariantStudio softwares.

To confirm and evaluate the putative disease causing genetic variations we designed primers by Primer3Plus software and executed PCR amplification for all members of our cohort [15]. PCR products were purified using High Pure PCR Product Purification Kit, (Roche) and sequenced on Applied Biosystems HITACHI 3500 Genetic Analyzer. For visualization of the Sanger sequencing we used FinchTV (Geospiza Inc.).

We compared the results with the following public databases: HGMD [16], LOVD [17], 1000Genomes.org [18], Alport database (ARUP) [19]. All the previously unpublished mutation were submitted in LOVD database (http://grenada.lumc.nl/).

In this study a genetic variant pathogenicity was concluded if the observed mutation:

1. was published previously or found in mutation databases
2. was not found among controls
3. it is not a common allele found in either dbSNP or in Exome Variant Server or in the scientific literature
4. its exclusive presence among affected family members was genetically proven.

We have not judged pathogenicity of any mutation by prediction programs.

## Results

### AS/FBH Multiplex AmpliSeq panel

Our AS/FBH AmpliSeq panel is designed for standard (not formalin-fixed paraffin embedded (FFPE)) DNA, with amplicon size range of 125–225 bp. The designed 197 amplicons were organized into two (99 and 98) pools. Our targets (all three genes coding exonic regions with 15 bp padding) were as follows: 5,013 bp *COL4A3* (covering 96.361% of the planned region), 5,073 bp *COL4A4* (97.02% covering) and 5,383 bp *COL4A5* (98.86% covering). The overall target size was 29.4 kb with an average coverage of 97.63%.

### Analysis of Hungarian non AS/FBH cohort

By analyzing the 66 Hungarian non AS/FBH controls we wished to identify population-specific polymorphisms in order to help distinguish polymorphisms from causative mutations in our AS/FBH cohort. We have found 21 unique non-synonymous exonic and intronic genetic variances within the investigated *COL4A3*, *COL4A4* and *COL4A5* genes. We identified 11 genetic variants in *COL4A3*, 9 variants in *COL4A4* and only one in *COL4A5* gene. All of them were already published elsewhere (Table 2).

**Table 2:** List of the identified polymorphisms in the investigated non AS/FBH cohort.

Gene	Variant	MAF	Consequence	Published in	Family (F) or Individual (I)
***COL4A3***	c.127G>C	0.153	p.Gly43Arg	[20]	F1 (M), F5 (M), F6 (M), F10 (M), F13 (M)
	c.1721C>T	0.121	p.Pro574Leu	[21]	F1 (M), F5 (M), F2 (F), F4 (F), F8 (F), F10 (M), F11 (F), F12 (F), F13 (M), F14 (F), I1(M), I2 (M), I4, F1 (M), F5 (M)
	c.3325C>T	0.024	p.Pro1109Ser	[22]	F4 (F)
	c.547-9A>C	0.024	IV	rs55667591	F3 (F), F4(F)
	c.3807C>A	0.040	p.Asp1269Glu	[21]	F2 (F), F6 (M)
	c.976G>T	0.226	p.Asp326Tyr	[23]	F3 (F), F6 (M), I3 (M), F7 (M), F13 (M), F11 (F), F8 (F)
	c. 2384-5T>C	0.113	IV	[24]	F10 (M), F7 (M)
	c.485A>G	0.798	p.Glu162Gly	[25]	F3 (F), I1 (F), F6 (M), F7 (M), F11 (F), F9 (F), F1 (M), F2 (F), F10 (M), F4 (F), I2 (M), F5 (M), F12 (F), F12 (F), I3 (M), F8 (F), F13 (M), F13 (F)
	c. 144+12C>A	0.339	IV	[25]	F8 (F) F3 (F), F10 (M), I1 (F), I2 (M), F6 (M), I3 (M), F13 (M), F11 (F), F9 (F)
	c.422T>C	0.798	p.Leu141Pro	[22]	F1 (M), F2 (F), F10 (M), F4 (F), I2 (M), F5 (M), F12 (F), F12 (F), I3 (M), F8 (F), F13 (M), F13 (F), F3 (F), I1 (F), F6 (M), F7 (M), F11 (F), F9 (F)
	c. 1352A>G	0.105	p.His451Arg	[21]	I2 (M), F9 (F)
***COL4A4***	c.2717-5A>T	0.040	IV	[26]	F11(F)
	c.2501A>G	0.008	p.Lys834Arg	[27]	F11 (F)
	c. 4207T>C	0.355	p.Ser1403Pro	[28]	F2 (F), F3 (F), I1 (F), I2 (M), F5 (M), F12 (F), F6 (M)F10 (M), F4 (F), F12 (F), F7 (M)
	c.3817+9G>C	0.363	IV	[29]	F1 (M), F10 (M), F4 (F), I3 (M), F7 (M), F13 (F)
	c.3979G>A	0.363	p.Val1327Met	[20]	F1 (M), F10 (M), F4 (F), F12 (F), I3 (M), F7 (M), F13 (F)
	c 1444C>T	0.435	p.Pro482Ser	[20]	F2 (F), F10 (M), I1 (F), I2 (M), F12 (F), F7 (M) F1 (M), F3 (F), F5 (M), F12 (F), F6 (M), F13 (F), F11 (F)
	c.3011C>T	0.444	p.Pro1004Leu	[20]	F2 (F), F3 (F), F10 (M), I1 (F), I2 (M), F5 (M), F12 (F), F6 (M), F7 (M), F1 (M), F4 (F), F12 (F), I3 (M), F13 (M), F13 (F)
	c.2996G>A*	0.008	p.Gly999Glu	[30]	F4 (F)
	c.4394G>A**		p.Gly1465Asp	[11]	F3(F)
***COL4A5***	c.2768-11A>G	0.065	IV	[31]	F3 (F), I1 (F), F10 (M), I2 (M), I3 (M)

minor allele frequency; IV: intronic variant; F: female; M: male)

* variant effect remained uncertain since we have found it in a control person (age 13 with two negative urinal samples, but we could not rule out later manifestation of FBH).

** this is a rare genetic variant with uncertain consequences. It was published both as polymorphism and as pathogenic mutation. In Family 3 this variant is co-segregating with a known pathogenic mutation (c.2320G>C), so we can not elaborate on the consequence of this variant when it is occurring separately.

### Variation classification workflow

**Fig 1:**
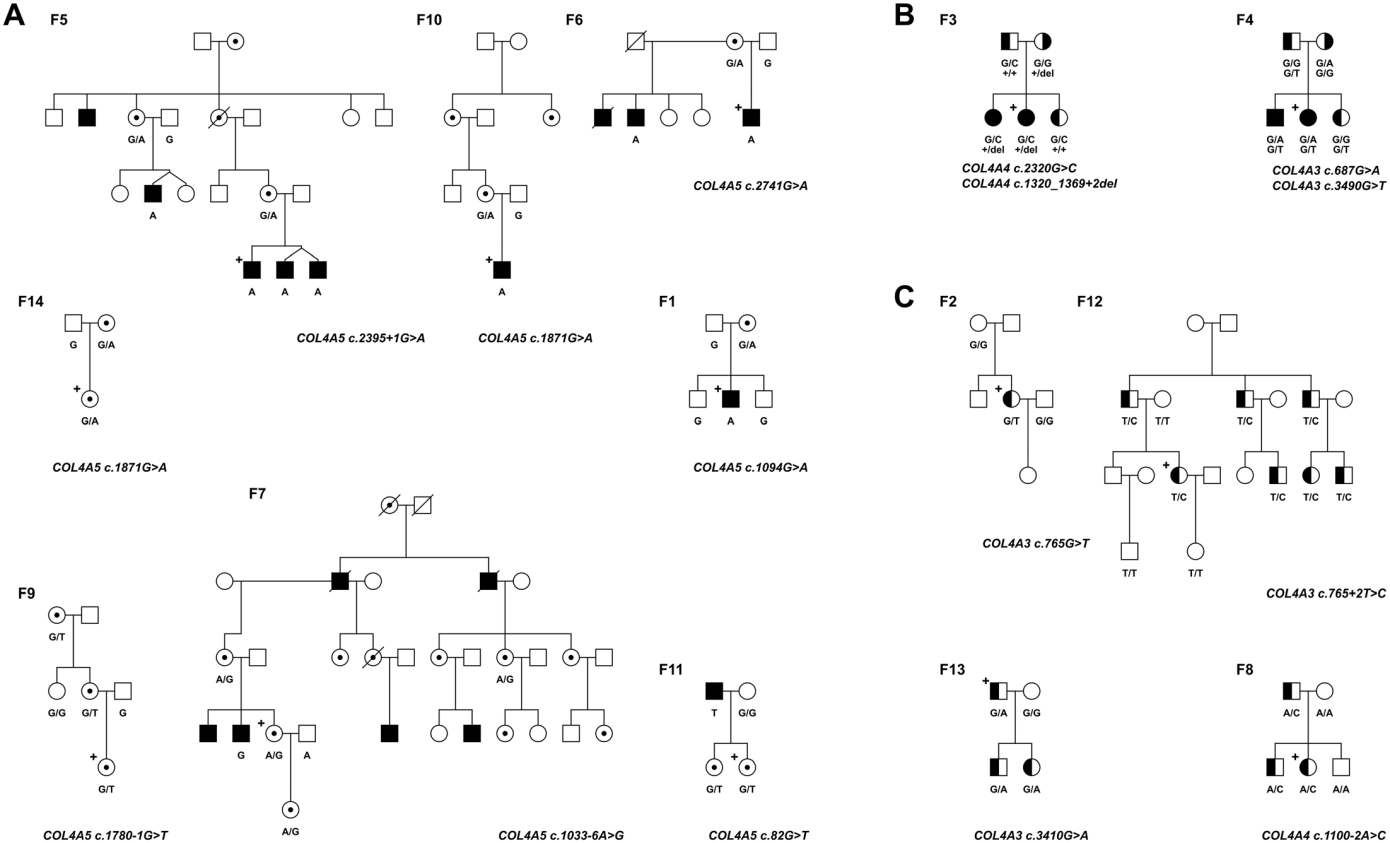
Pedigree of investigated AS/FBH Hungarian families. A, Families with X-linked AS. B, Families with autosomal AS. C, Families with FBH. Open squares indicate males and open circles indicate females. An oblique bar indicates a deceased individual. White symbols indicate individuals without clinical sings of the AS/FBH disease. Filled black symbols denote individuals with hematuria and/or proteinuria, hypoacusis or renal failure. The segment shaded circles and squares indicate carriers for a Chromosome 2 (*COL4A3* or *COL4A4*) mutation. A circle that has a dot inside indicates a *COL4A5* mutation carrier. The plus signs indicate the index patients whose DNA was analyzed by NGS. The identified mutation(s) for each family is shown.

The available medical records and the predictive clinical and pathological results are often incomplete or not conclusive enough to distinguish between FBH and the different types of Alport (autosomal or X-linked), especially in not-so-large families (see Table 1 and Fig 1) Therefore, we decided to do sequencing-based genetic analysis on the Hungarian AS/FBH cohort using our AmpliSeq panel. Selection criteria for individuals for the initial NGS analysis were as detailed in Methods. Then, as results of NGS analysis all the identified unique 37 genetic alterations were compared to data of publicly available databases such as LOVD, dbSNP, HGMD and Alport database (University of Utah). As a result we identified the 21 published, previously mentioned polymorphisms (Table 2) and 4 published mutations (2 in *COL4A4* and 2 in *COL4A5* genes). All the four published mutations were validated in the appropriate AS/FBH cases, where we confirmed that only affected individuals carried them. The 14 remaining alterations were investigated among AS/FBH family members. We have classified all of these alterations as new mutations (5 in *COL4A3* gene; 3 in *COL4A4* and 6 in *COL4A5* genes) as their exclusive presence was confirmed in the affected family members by Sanger sequencing (see individual family trees in Supplements). Six out of the thirteen SNP mutations were amino acid change (missense mutations), while the remaining seven SNP mutations were found in the splice recognition sites (splice variants). We also identified a *COL4A4* mutation affecting part of exon 20 and 2 bases in the splice donor site that is a 52 bp deletion shortening the exon and altering splicing (Table 3).

**Table 3:** List of the identified mutations in our Hungarian AS/FBH cohort.

Gene	Variant	Consequence	Family/Individual	Published in
***COL4A3***	c.687G>A	splice region	F4(F)	this paper
	c.3490G>T	p.Gly1164Cys	F4(F)	this paper
	c.765+2T>C	splice donor	F12(F)	this paper
	c.3410G>A	p.Gly1137Asp	F13(M)	this paper
	c.765G>T	splice region	F2(F)	this paper
***COL4A4***	c.2320G>C	p.Gly774Arg	F3(F)	[32]
	c.2986G>A	p.Gly996Arg	I3(F)	this paper
	c.1100-2A>C	splice acceptor	F8(F)	this paper
	c.1320_1369+2del	deletion, splice donor	F3 (F)	this paper
***COL4A5***	c.1780-1G>T	splice acceptor	F9(F)	this paper
	c.1871G>A	p.Gly624Asp	F10(M), F14(F), I2(M)	[33]
	c.1033-6A>G	presumed splice variant	F7(M)	this paper
	c.2395+1G>A	splice donor	F5(M)	this paper
	c.1094G>A	p.Gly365Glu	F1(M)	[34]
	c.2741G>A	p.Gly914Asp	F6(M)	this paper
	c.82G>T	p.Ala28Ser/ splice acceptor	F11(F)	this paper
	c.1010G>T	p.Gly337Val	I1(M)	this paper

(F: female; M: male)

To demonstrate the ease and usefulness of our methodology, we categorized all of the 14 families as follows: F1, F5-7, F9-11 and F14 have X-linked AS; F3 and F4 have autosomal semi-dominant AS; F2, F8, F12 and F13 have FBH (see Fig 1A–1C). We had no information about additional family members in the individual (I1-I3) cases, but using our genetic diagnostic approach we could categorize them as X-linked AS (I1 and I2) and FBH (I3).

## Discussion

AS and FBH are inherited renal diseases, with an incidence rate of 1:50,000 in case of AS and around 1:200 in case of FBH [33,35]. Type IV collagen is an important structural protein in basement membranes, has a triple helical structure, which consists of a Col4A3, a Col4A4 and a Col4A5 protein [36]. Since *COL4A3* and *COL4A4* genes are coded in an autosomal chromosome, a single mutation in any of these two genes only leads to FBH in both sexes, as half of the triple helix has normal structure. However, a single mutation in X chromosome coded Col4A5 protein will lead to AS in males (disrupting all forming triple helix) and varying, intermediate phenotype in females. Genetically FBH is a special carrier form of AS since it also causes phenotype (not like in classical recessive metabolic diseases such as PKU) and traditionally treated as independent disease. Successful identification of genetic mutation in any of these 3 *COL4A* genes—even before the clinical signs can be observed—could provide an early differential diagnosis for FBH/Alport patients.

We established a new, amplicon based NGS method which has three advantages: 1, it is reliable, cheaper (less than 500 euro per sample) and faster than the traditionally used Sanger capillary sequencing, 2, can provide DNA sequence information from all three *COL4A* genes simultaneously and 3, has the potential to detect single nucleotide polymorphisms (SNPs) and various sizes of insertions/deletions as well. Using our NGS panel and the approach outlined in this paper we successfully identified causative mutations in all investigated AS/FBH instances.

In our study 37 genetic variations were identified from which there are 14 unpublished and 3 published mutations, thus causative mutations were successfully identified in all investigated cases. Out of our 17 cases 10 (as was expected) were X-linked AS (caused by 8 different *COL4A5* mutations), we had 5 FBH cases (3 with three different *COL4A3* and 2 with two different *COL4A4* mutations). In case of Family 4 we have identified two *COL4A3* mutations, while in Family 3 we have identified two *COL4A4* mutations causing autosomal AS in both cases. In our cohort we not only detected non-synonymous (amino acid change) SNPs and splice mutations, nine and seven respectively, there was also a 52 bp deletion revealed. Eight amino acid changes caused Glycine substitution in collagenous domains, what is a well-known pathogenic alteration in Col4A proteins. Interestingly, a missense mutation (c.82G>T; p. Ala28Ser mutation in family 11, which also alters a conserved base of the splice acceptor site) was detected in a non-collagenous domain of Col4a5, which segregation was coherent with the observed clinical symptoms in the family. In two other cases the splice regions within exons, in two cases splice donor sites, while in 3 cases splice acceptor sites were affected. There was a *COL4A5* splice site mutation variant which is outside the usual +/- 1–2 splice donor/acceptor site (c.1033-6G>A in Family 7), that although fulfilled all of our mutation classification criteria (detailed in Material and Methods) without additional functional test we only presumed that it is pathogenic.

Up to now there are less than 1000 classified causative variants in public databases (HGMD, dbSNP, ClinVar) regarding the three *COL4A* genes causing AS/FBH which are around 4% of the whole exonic target region. The vast majority of the published mutations coming from X-linked *COL4A5* data, even if either of the two autosomal *COL4A* genes is longer and their mutations can cause not only the more frequent FBH but also AS as well. One of the possible explanations could be that lots of FBH cases (caused by a single mutation either of *COL4A3* or *COL4A4*) remain undiagnosed. With the introduction of simultaneous sequencing of all three *COL4A* genes by NGS, we should expect more sequence data from *COL4A3* and *A4* in the near future. Both mutations and polymorphisms in the 3 investigated *COL4A* genes are thought to be highly population-specific (due to non-existing selection pressure in case of the polymorphisms and only low selection pressure in case of FBH). But, to our surprise, none of the 21 identified genetic variations in our control group proved to be unpublished and Hungarian population specific. In scientific literature an autosomal dominant (AD) form of AS is also mentioned. Most of the autosomal dominant AS data comes from an era where the technical limit (error rates) of sequencing was comparable to the claimed frequency (around 1%) of this form of AS. However, a recent paper using NGS claims that there was an astonishing portion of autosomal dominant AS (31%) of their solved cases [10]. In the light of these data we should expect to find around 5 AD AS among our 17 cases, but we have not found any.

In our opinion there is a widespread confusion in the clinical and pathological terminologies used to describe FBH, Thin Basement Membrane Nephropathy (TBMN) and AS. FBH is used by nephrologist as a descriptive term for cases when only haematuria (usually non progressive, without proteinuria or impaired hearing or vision) could be detected throughout the family, which is only a subset (although the largest) of all the TBMN cases. TBMN on the other hand is a term used by pathologists describing the thin basement membrane phenomenome of the kidney, which in some cases result slowly progressive haematuria with proteinuria and as an age-related process can reach ESRD in elderly patients. In their paper Fallerini C. et al presented 7 AD AS families in detail with a single identified autosomal *COL4A3* or *A4* mutation [11]. Their conclusion could be biased by the fact that 45% of their AS/FBH cases went without mutation identification. It is also worth to note that in all of their published AD AS cases ESRD presented only in older age (between 52–74 with an average of 63 years), without a single case with eye problem and with only 4 elderly cases with hearing loss. According to WHO data (updated in 2015 march) approximately one-third of people over 65 years of age are affected by disabling hearing loss which is fully agrees with their reported rate.

Further on there is no known explanation for the biological foundation of AD AS. In cases where collagen triple helix formed by one (as in case of Type VII collagen, 3 identical chains coded by *COL7A1* gene) or two different collagen chains (as in case of Type I collagen, consisting of two chains coded by *COL1A1* and one coded by *COL1A2*) the dominant negative effect of a single autosomal mutation is known [37,38]. In these cases, due to the formation of homodimers/homotrimers, a single mutation in an autosomal gene could lead to stronger phenotype than a null allele. The mutated protein will bind to the normal protein and result only around 25% of functional dimer (and even less in case of trimers) while autosomal null alleles lead to ~50% functional protein in heterozygotes. This is not the case in the forming Col4a3-4-5 triple helix, where all three chains are different and coded by different genes, so it does not comply with the current biological model of negative dominant effect. FBH biologically is the result of reduced amount of proper Col4a3-4-5 triplex, while AS is the result of the lack of the functional protein.

Based on our own observation on the genetic analyses presented here; confusing interpretation on age related clinical symptoms and the lacking of biological foundation we think that AD AS as genetic term doesn’t exist and shouldn’t be used. We think that the alleged autosomal Alport cases genetically are either a single severe autosomal mutation leading to phenotype in between FBH/AS only in higher age (so it is a slowly evolving TBMN) or it could also be the result of unsuccessful genotyping of autosomal AS due to technical (for example the use of ineffective insertion/deletion detection algorithm) or differences in their genetic variation classification approach that heavily relies on prediction softwares.

As far as we see Alport syndrome and its related diseases and their observed clinical/pathological heterogeneity (heamaturia, ESRD, hypoacusis, TBMN) is such a biological problem, which has quantitative inheritance elements. In individuals without mutations in the three investigated *COL4A* genes has the full amount of fully functional triple-helix. In the individuals (independently from gender) with a mutation in either *COL4A3* or *COL4A4* gene there is about half of the theoretical maximum amount of functional triple-helix. If there is a *COL4A5* mutation in a male, than there isn’t any fully functional triple-helix formed. In females, since random inactivation of X chromosome occurs, approximately 50% of fully functional triple-helix will form, but this could be altered (positively or negatively) by the imbalances of X inactivation. This also could explain the variable phenotype even in a family. The situation in the case of extracellular basal membrane is even more complex, since the observed clinical phenotype does not depend on a single cell genotype rather from the genotype ratio within Type IV collagen producing cell population. The other sources of the heterogenity naturally are the mutations causing different level of malfunction in the forming triple-helix. This phenotypic variablity leads to introduce the term semi-dominant inhertance, which has an intermediate phenotype different from both dominant (since the phenotype of a heterozygote is not the same as a homozygote mutant) or recessive (since the phenotype of a heterozygote is not the same as a homozygote normal). As a result of this we can view Alport and its related diseases as a spectrum disorder, where the biological base of the different phenotypes is the amount of fully functional (or impared/null) Type IV collagen formed. The two extremities of this spectrum are FBH with only mild, non-progressing haematuria, while the other end is Alport syndrome with serious kidney, eye and hearing phenotype.

Because the prognosis of FBH and AS is entirely different while the early symptoms could be similar, both the genetic classification and the above mentioned distinction in our view is extremely important. By providing correct genetic data and by establishing the correct inheritance pattern within family, it could help both clinicians and genetic counselors 1) to communicate and make an early, informed decision about the possible speed and direction of the progression in the family, 2) to establish the circle of the family members who need regular checking, 3) to select a possible kidney donor within the family and 4) support positive family planning. The genetic test could lead to the earliest diagnosis even before the manifestation of clinical symptoms that would be a big benefit for patients, for their families and for the health systems as well. For patients with some of the earlier clinical symptoms or even before, genetic diagnosis could mean preventive treatment such as using ACE inhibitors which could delay the onset of end-stage renal failure and improve life expectancy, even when begun before the onset of proteinuria [5,6,26]. For family members the biggest benefit could be the possibility for positive family planning, and the awareness of possible kidney problems. And for the health system the cheap genetic diagnostic procedure eliminates the need for long and uncertain clinical staying, and lots of unnecessary, less conclusive and much more expensive tests and examinations. The best tool for this job is the DNA-based molecular genetic analysis of the proband and his/her relatives. Using peripheral blood as DNA source for genetic analysis causes much less trauma for patient (especially children) compared to the kidney biopsy, which carries certain level risk of severe complications [27]. The result of the DNA sequencing is independent of the age of the proband, from the stage of the disease and in most cases provides quick and accurate confirmation of the diagnosis. It can be also performed on a single person independently when the family history is not available. Furthermore, in cases where no detailed clinical and pathological data is available genetic analyses could be the first choice.

We believe that our classification approach and the presented genetic data could contribute to better, faster and cheaper diagnosis of AS/FBH patients and their relatives. In order to further facilitate the mutation detection rate in AS/FBH our AmpliSeq panel was offered as publicly available panel (Ion Community Panel) in the AmpliSeq Designer site (www.ampliseq.com) and see also S1 File.

## Supporting Information

S1 File Our custom AmpliSeq Alport-panel design files. (ZIP)
